# Calendar

**DOI:** 10.1155/EDR.2001.166

**Published:** 2001


					166
CALENDAR
October 5-7, 2001
4th Annual Diabetes
Conference
Destin, FL, USA
Contact: Education
Department
Tel: 1-800-423-4992
Fax: +1-205-945-1548
e-mail: pwilliams@sma.org
October 29 November 01,
2001
International Conference on
Recent Advances on Diabetes
Mellitus
Ajman, United Arab Emirates
Contact: Prof. J Shanmugam
Tel: 9716-7431-333
Fax: 9716-743-222
e-mail: cs@gmcajman.com
October 30- 31, 2001
1st National Diabetes
Facilitators Group Conference
Weston Super Mare, UK
Contact: NDFG Conference
Secretariat
Tel: +44-0-1794 511332
Fax: +44-0-1794 511455
e-mail: icms@dial.pipex.com
November 10, 2001
Frontiers in Diabetes Research:
3rd Annual Conference
New York, NY, USA
Contact: Center for Continuing
Education, Columbia University
College of Physicians and
Surgeons, 630 West 168th,
Street, Unit 39, New York, NY
10032
Tel: +1-212-305-3334
Fax: +1-212-781-6047
e-mail: cme@columbia.edu
November 10,2001
Diabetes Symposium
Hershey, PA, USA
Contact: Continuing
Education, Penn State College
of Medicine, P.O. Box 851,
Hershey, PA 17033
Tel: +1-717-531-6483
Fax: +1-717-531-5604
e-mail: plinton@psu.edu
March 26-29, 2002
6th Pan Arab Conference on
Diabetes: PACD 6
Cairo, Egypt
Contact: Dr. Mahmoud Ashraf
Mohamed
Tel: 20-122-131-868
Fax: 2-022-723-693
e-mail:
mahmoud@arab-diabetes.com
January 10-16, 2002
Diabetes Mellitus: Molecular
Mechanisms, Genetics and New
Therapies (J4)
Keystone, CO, USA
Contact: Keystone Symposia,
Drawer 1630, 221 Summit
Place Suite 272, Silverthorne,
CO 80498
Tel: 1-800-253-0685
+1-970-262-1230
Fax: +1-970-262-1525
e-mail:
inf6@keystonesymposia.org
March 20-22, 2002
5 Congresso Portugus de
Diabetes
Vilamoura-Mariontel,
Portugal
Contact: K.I.T., a/c Sociedade
Portuguesa de Diabetologia,
Pra.a MarquSs de Pombal, 16A
5 Piso
Tel: +351-213-504064
Fax: + 351-213-504024
e-mail: llouro@kit.de
June 15-18, 2002
62nd Annual Scientific Sessions
of the American Diabetes
Association
San Francisco, CA, USA
Contact: ADA's Meetings
Department
Tel: +1-703-549-1500 ext.3553
e-mail: meetings@diabetes.org
September 1-5, 2002
Annual Conference of the
European Association for the
Study of Diabetes
Budapest, Hungary
Contact: Meeting Organizer
Tel: 49-0-6050-97220
Fax: 49-0-6050-972-210
e-mail: PS@profi-sales.com
INTERNATIONAL JOURNAL OF EXPERIMENTAL DIABETES RESEARCH

				

